# Association Between Resident Physicians’ Self-Rated Patient Care Ownership and Medical Professionalism Assessed by Patients: A Single-Center Study

**DOI:** 10.7759/cureus.94413

**Published:** 2025-10-12

**Authors:** Hirohisa Fujikawa, Takuya Aoki, Daisuke Son, Masato Eto

**Affiliations:** 1 Department of General Medicine, Juntendo University Faculty of Medicine, Tokyo, JPN; 2 Center for General Medicine Education, School of Medicine, Keio University, Tokyo, JPN; 3 Department of Medical Education Studies, International Research Center for Medical Education, Graduate School of Medicine, The University of Tokyo, Tokyo, JPN; 4 Division of Clinical Epidemiology, The Jikei University School of Medicine, Tokyo, JPN; 5 Section of Clinical Epidemiology, Department of Community Medicine, Graduate School of Medicine, Kyoto University, Kyoto, JPN; 6 Department of Community-Based Family Medicine, School of Medicine, Tottori University Faculty of Medicine, Tottori, JPN

**Keywords:** patient, patient care ownership, professionalism, residency, resident

## Abstract

Introduction

Patient care ownership (PCO) has received substantial attention as a core aspect of medical professionalism. In recent years, a quantitative PCO Scale (PCOS) has been developed and widely utilized. Despite its growing importance, PCOS is measured through resident physician self-assessment, and the association between PCOS scores and resident evaluation by patients, the primary stakeholders in clinical care, remains underexamined.

Methods

This study was conducted at a rural postgraduate clinical training hospital in Japan from July 2022 to March 2023. PCO was assessed using the Japanese version of the PCOS (J-PCOS) as the explanatory variable. Patient-reported medical professionalism was measured using the Japanese version of the Instrument for Patient Assessment of Medical Professionalism (J-IPAMP) as the outcome variable. A linear mixed-effects model was employed to adjust for clustering within residents and individual covariates.

Results

Twelve residents and 99 of their patients were included in the analysis. After adjusting for potential confounders and clustering within physicians, J-PCOS scores were not associated with J-IPAMP scores.

Conclusions

Residents’ self-rated PCO was not associated with patient-reported professionalism. This may be because patients cannot observe key aspects of PCO, such as interprofessional communication and decision-making, and instead evaluate professionalism based on visible behaviors, such as bedside manner. Differences in priorities, with patients focusing on relational aspects of care while physicians emphasize systemic responsibilities and outcomes, may also contribute to this disconnect. Future studies should employ a multicenter design in Japan and conduct analogous surveys internationally.

## Introduction

There has been growing global interest in medical professionalism [1]. It is essential for maintaining public trust in healthcare [2] and has been shown to influence physician-patient relationships, quality of care, and ultimately health outcomes [3]. Therefore, fostering medical professionalism among physicians is crucial for ensuring optimal patient care [1].

Recently, patient care ownership (PCO) has received considerable attention as a key component of medical professionalism [4]. PCO refers to the affective-cognitive state arising from a physician’s knowledge and management of patients, as well as their emotional investment in patient relationships [5-7]. It is expected that strong PCO enhances clinical skills and improves patient care quality [5,8,9], making its cultivation during residency particularly important.

Traditionally, PCO has been described as “the philosophy that one (i.e., the doctor) knows everything about one’s patients and does everything for them” [10]. However, recent research has expanded the concept to encompass multiple dimensions, including physician actions (advocacy, communication and care coordination, therapeutic decision-making, follow-through, acquiring knowledge of the patient, and leadership), physician attitudes (doing more than the minimum requirement, viewing oneself as ultimately responsible, and feeling accountable for patient care and outcomes), physician identity (serving as the primary or main care provider), physician qualities (taking initiative), and quality of patient care (ensuring comprehensive, longitudinal, and patient-centered care) [4].

Previous research on PCO was largely qualitative. In 2019, the quantitative PCO Scale (PCOS) was developed in the US, and its reliability and validity were confirmed in multicenter studies [11]. A Japanese version of the PCOS (J-PCOS) was developed in 2021 and shown to have acceptable reliability and validity among Japanese medical residents [12]. This scale is expected to be useful in a variety of settings, including educational program research aimed at fostering ownership and exploratory studies on the impact of ownership on healthcare outcomes [11,12].

Nevertheless, PCOS is measured through resident self-assessment, and the association between PCOS scores and patient evaluation of residents, arguably the most important stakeholders in clinical care, remains unknown. Our research question was therefore as follows: Is residents’ self-rated PCO associated with medical professionalism as evaluated by patients?

## Materials and methods

Study design and participants

From July 2022 to March 2023, we conducted a cross-sectional study at a postgraduate clinical training hospital, a community hospital located in a rural area of Japan. Residents in postgraduate years (PGYs) 1-5 who were enrolled in the hospital’s clinical training program during the study period were eligible. Residents who declined participation were excluded. Patients were eligible if they were inpatients aged 20 years or older under the care of participating residents during the survey period. Patients were excluded if they were unable to complete the questionnaire due to severe physical or mental disorders or if they declined participation.

Questionnaires for medical residents were distributed via the training administrator, while those for patients were distributed by receptionists or survey staff. Patients completed the questionnaire, placed it in an envelope, and submitted it to a collection box at the hospital. Both residents and patients were informed that participation was voluntary and that responses would be accessible only to the researchers. Informed consent was obtained from all participants.

Outcome variable: instrument for patient assessment of medical professionalism

Patient medical professionalism was assessed using the Japanese version of the Instrument for Patient Assessment of Medical Professionalism (J-IPAMP) [13] (Appendix A). The original IPAMP was developed by Ratelle et al. in 2020 [14], building on earlier instruments by Dine et al. and the American Board of Internal Medicine, which demonstrated good internal consistency, reliability, and construct validity [15-17]. Ratelle et al. iteratively refined the instrument, confirming its acceptable reliability and validity [14]. The J-IPAMP, developed in 2021, has also demonstrated sufficient reliability and validity [13]. It is an 11-item tool scored on a 5-point Likert scale (1 = poor, 2 = fair, 3 = good, 4 = very good, and 5 = excellent), giving a total score range of 11-55, with higher scores indicating greater patient-assessed medical professionalism [13].

Explanatory variable: PCOS

Residents’ PCO was assessed using the Japanese version of the PCOS (J-PCOS) [12], a 13-item questionnaire (Appendix B). Each item is scored on a 7-point Likert scale (1 = strongly disagree to 7 = strongly agree), giving a total score range of 13-91, with higher scores indicating higher levels of PCO [12]. The J-PCOS score was used as the explanatory variable.

Covariates

Based on prior research [11,12,14,18], we selected covariates potentially associated with PCO and patient-assessed professionalism. These included residents’ sex, PGY level, and specialty.

Statistical analysis

Data consisted of patients nested within physicians, suggesting the need for multilevel modeling due to the hierarchical structure. Accordingly, we used a linear mixed-effects model (random intercept model) with random effects for physicians and fixed effects for covariates (residents’ sex, PGYs, and specialty). Statistical assumptions for the linear mixed-effects model were checked and met prior to analysis.

The sample size was determined based on feasibility, including all PGY 1-5 residents training at the hospital during July 2022-March 2023 and all inpatients aged ≥20 years under their care during the survey period. We conducted a complete case analysis, including only resident-patient dyads with no missing data. A dyad was considered complete when the dependent variable (patient-rated professionalism), explanatory variable (resident self-rated PCO), and all covariates were observed. Statistical significance was set at a two-tailed p-value < 0.05. Analyses were performed using IBM SPSS Statistics for Mac, Version 29.0 (Released 2022; IBM Corp., Armonk, NY, USA).

Ethical considerations

The study was approved by the Institutional Review Board of the University of Tokyo (2021074NI).

## Results

Twelve of the 13 eligible medical residents agreed to participate in the study. Of the 105 patients who consented and completed the questionnaire, six had missing data. Consequently, 99 patients were included in the analysis. Participants’ characteristics are summarized in Table 1.

**Table 1:** Characteristics of the participants J-PCOS scores range from 7 to 91, with higher scores indicating greater PCO. J-IPAMP scores range from 5 to 55, with higher scores indicating a higher level of medical professionalism as assessed by patients. J-IPAMP, Japanese version of the Instrument for Patient Assessment of Medical Professionalism; J-PCOS, Japanese version of the Patient Care Ownership Scale; PCO, patient care ownership

Characteristic	Value
Physicians	
Sex, n (%)	
Female	4 (33.3)
Male	8 (66.7)
Others	0 (0.0)
PGYs, n (%)	
1	2 (16.7)
2	1 (8.3)
3	3 (25.0)
4	2 (16.7)
5	4 (33.3)
Specialty, n (%)	
Internal medicine	10 (83.3)
Orthopedics	2 (16.7)
J-PCOS, mean (SD)	64.7 (8.9)
Patients	
Sex, n (%)	
Female	46 (46.5)
Male	53 (53.5)
Others	0 (0.0)
Age group, n (%)	
20-24 years	1 (1.0)
25-34 years	3 (3.0)
35-44 years	5 (5.1)
45-54 years	13 (13.1)
55-64 years	18 (18.2)
65-74 years	28 (28.3)
≥75 years	31 (31.3)
Education, n (%)	
Less than high school	10 (10.1)
High school	45 (45.5)
Junior college	27 (27.3)
College or higher	17 (17.2)
Duration of hospitalization, n (%)	
1-10 days	64 (64.6)
11-20 days	19 (19.2)
21-30 days	8 (8.1)
31-40 days	3 (3.0)
41-50 days	1 (1.0)
51-60 days	2 (2.0)
≥61 days	2 (2.0)
J-IPAMP, mean (SD)	41.7 (8.9)

Table 2 presents the results of the multilevel analysis examining the association between J-PCOS and J-IPAMP scores. After adjusting for potential confounders and clustering within physicians, J-PCOS scores were not significantly associated with J-IPAMP scores (adjusted mean difference per 1-point increase in J-PCOS, -0.05; 95% CI: -0.27 to 0.18).

**Table 2:** Association of J-PCOS with J-IPAMP The random intercept model was adjusted for resident physicians’ sex, PGYs, and specialty. J-IPAMP, Japanese version of the Instrument for Patient Assessment of Medical Professionalism; J-PCOS, Japanese version of the Patient Care Ownership Scale; PGY, postgraduate year

Unadjusted mean difference	95% CI	Adjusted mean difference	95% CI
-0.03	-0.26 to 0.21	-0.05	-0.27 to 0.18

## Discussion

Our study indicated that there was no significant association between scores of the J-PCOS and J-IPAMP. These findings suggest a discrepancy between PCO as assessed by residents themselves and as perceived by their patients.

Several studies have reported a perception gap between healthcare professionals and patients. A Canadian cross-sectional study demonstrated that doctors and patients held differing perspectives on doctors’ communication skills during clinical encounters [19]. Similarly, a German study found that physicians’ perceptions of healthcare service quality substantially differed from those of patients and other employee groups [20]. To our knowledge, however, no prior studies have compared residents’ self-evaluated performance with patient-assessed evaluations. Our findings underscore the importance of assessing medical residents from multiple stakeholder perspectives, particularly patients.

The lack of association between PCO and professionalism assessed by patients may be explained by several factors. First, the two constructs do not necessarily measure the same behavioral domains. Core elements of PCO, such as advocacy, interprofessional communication, and behind-the-scenes decision-making, are often not directly observable by patients. Patients are more likely to evaluate professionalism based on visible interactions, such as bedside manner [21]. Second, patients and physicians may prioritize different aspects of care: patients may emphasize relational components, while physicians focus on systemic responsibilities and outcomes [22]. High-context cultures, which are predominant in Japan and other Asian countries, rely heavily on indirect and nonverbal communication [23]. In such settings, these differences in priorities may remain unspoken, perpetuating divergence. Additionally, in Japan, cultural values such as courtesy and harmony may lead patients to focus more on relational aspects when assessing professionalism [24]. Future research could explore how patient and resident perspectives align or diverge in evaluating professionalism and ownership of patient care.

We note three limitations of our study. First, this was a single-center study, so selection bias cannot be excluded. Conducted in a rural community hospital, the characteristics of residents and patients may differ from those in urban or university hospitals, potentially affecting questionnaire responses [25]. Future studies should examine this research question across multiple institutions with diverse settings. Moreover, the limited sample size may have reduced statistical power, meaning the null result should be interpreted as inconclusive rather than evidence of no association. Second, the study occurred during the COVID-19 pandemic. Residents may have limited direct patient interaction due to infection-control policies, shifting aspects of care to team interactions outside patients’ view. This context could diminish patients’ ratings of relational behaviors and obscure the visibility of ownership behaviors, further weakening any observable association with PCO. Third, although covariates were selected based on previous literature, unknown confounders may have influenced the results. For instance, patient disease severity and treatment outcomes could affect both resident behavior and patient evaluations.

Despite these limitations, two key implications emerge. First, evaluation of residents’ PCO should employ a multi-source, multi-method approach. Medical educators should triangulate self-rated PCO with patient-reported professionalism, 360-degree evaluations from other healthcare professionals [26], and structured direct observation. Second, PCO is influenced by cultural and systemic contexts; what is visible and valued varies across settings [27]. Future research should therefore include multicenter studies within Japan and similar studies internationally to facilitate cross-cultural comparison. Such evidence will enhance our understanding of residents’ PCO and inform strategies to foster professionalism across diverse healthcare environments.

## Conclusions

Our study showed that J-PCOS scores were not associated with J-IPAMP scores. This lack of association may reflect patients’ limited ability to observe many aspects of PCO, such as interprofessional communication and decision-making, leading them to evaluate professionalism based primarily on observable interactions, like bedside manner. Additionally, differences in priorities, with patients emphasizing relational aspects of care while physicians focus on systemic responsibilities and outcomes, may contribute to this disconnect. Future research should include multicenter studies within Japan, as well as comparable studies internationally, to better understand these dynamics.

## Appendices

Appendix A

**Table 3:** Questionnaire for patients (the Japanese version of the Instrument for Patient Assessment of Medical Professionalism)

Item	English version	Japanese version
1	How is this doctor at letting you tell your story; listening carefully; asking thoughtful questions; not interrupting you while you’re talking?	この医師は、あなたに自分の話をさせる、あなたの話に耳を傾ける、思いやりのある質問をする、あなたが話をしているときにさえぎらないという点では、いかがでしょうか。
2	How is this doctor at showing interest in you as a person; not acting bored or ignoring what you have to say?	この医師は、あなたを一人の人間として関心を示す、退屈そうにせず、あなたが伝えなければならないことを無視しないという点では、いかがでしょうか。
3	How is this doctor in treating you like you’re on the same level; never “talking down” to you or treating you like a child?	この医師は、あなたと同じ目線で接する、決して「上から目線」で話さない、子供扱いすることはないという点では、いかがでしょうか。
4	How is this doctor in greeting you warmly; calling you by the name you prefer; being friendly, never crabby or rude?	この医師は、あなたに温かく挨拶をする、呼んで欲しい名前で呼んでくれる、親しみやすい、決して不機嫌であったり失礼であったりはしないという点では、いかがでしょうか。
5	How is this doctor at telling you everything; being truthful, upfront, and frank; not keeping things from you that you should know?	この医師は、あなたに全てを話す、誠実、正直かつ率直である、あなたが知るべきことを隠さないという点では、いかがでしょうか。
6	How is this doctor at warning you during the physical exam about what he/she is going to do and why; telling you what he/she finds?	この医師は、身体診察の際に、何を、なぜしようとしているかを説明してくれる、何が分かったのかを伝えてくれるという点では、いかがでしょうか。
7	How is this doctor at using words you can understand when explaining your problems and treatment; explaining any technical medical terms in plain language?	この医師は、あなたの問題や治療について説明する際、あなたが理解しやすい言葉を使用する、専門的な医療用語をすべてやさしい言葉で説明するという点では、いかがでしょうか。
8	How is this doctor at respecting your thoughts and beliefs; putting himself/herself “in your shoes”?	この医師は、あなたの考えや信念を尊重する、あなたの立場に立って考えるという点では、いかがでしょうか。
9	How is this doctor at explaining what you need to know about your problems, how and why they occurred, and what to expect next?	この医師は、あなたの問題について知る必要があること、その問題がどのように、なぜ起こったのか、そして次に起こりうることを説明するという点では、いかがでしょうか。
10	How is this doctor at discussing options with you; asking your opinion; offering choices and letting you help decide what to do; asking what you think before telling you what to do?	この医師は、選択肢についてあなたと話し合いをする、あなたの意見を求める、選択肢を提示して何をすべきか決められるよう手助けしてくれる、何をすべきか伝える前にあなたの考えをたずねるという点では、いかがでしょうか。
11	How well did this doctor keep you informed about your plan of care; notifying you of upcoming tests and treatments?	この医師は、どの程度、治療計画についてあなたに情報を与え、どの程度、今後の検査や治療の予定をあなたに知らせましたか。

Appendix B

**Table 4:** Questionnaire for medical residents (the Japanese version of the Patient Care Ownership Scale)

Item	English version	Japanese version
1	I was vocal and assertive about my patients’ best treatment/care.	私は、担当患者にとっての最善の治療・ケアについて、積極的に自らの意見を述べました。
2	I was the “go-to” person for knowledge about my patients.	私は、担当患者について誰よりもよく知っていると周りに頼りにされました。
3	I was proactive in checking up on my patients, rather than being called with questions or concerns.	私は、質問や懸念について呼びかけられるよりもむしろ、先回りをして担当患者について確認していました。
4	I ensured good continuity of care even when I was absent from the service.	私は、私が不在のときでも、担当患者のケアがうまく継続されるよう努めました。
5	I felt comfortable telling the attending what I felt was the right thing to do for my patients, rather than just letting them decide.	私は、担当患者の診療方針について、上級医まかせにするのではなく、患者にとって最善だと私が思ったことを上級医に対して気兼ねなく伝えました。
6	I made sure that the nursing staff was updated with the day’s plan.	私は、担当患者の一日の計画について看護スタッフが最新の情報を把握しているか確認しました。
7	I was given the opportunity to make decisions independently about my patients’ care.	私は、担当患者のケアについて自ら決断を下す機会を与えられました。
8	I personally made sure to go back and check that all orders were actually carried out.	私は必ず、すべての指示が実際に行われたかどうか、さかのぼって自ら確認しました。
9	When carrying out my patient’s management plan, I took extra care to make sure that things did not fall through the cracks.	私は、担当患者の診療計画を実行する際、万が一なにか抜け落ちていることがないか確認するため十分に注意を払いました。
10	I felt comfortable making decisions independently about my patients’ care.	私は気兼ねなく、担当患者のケアについて自主的に決断を下しました。
11	I challenged the team as needed if I felt it was in my patients’ best interest, no matter how much push back I got.	私は、担当患者にとって最善の利益になると思えば、たとえ担当チームの反対に遭ったとしても必要に応じて異議を唱えました。
12	I felt responsible for my patients’ care, even after my shift ended.	私は、自分の勤務シフトの終了後も、担当患者のケアに関して責任を感じていました。
13	I felt a strong sense of ownership of my patients’ care.	私は、担当患者のケアに対して強い当事者意識を持っていました。
